# Examination of the xanthosine response on gene expression of mammary epithelial cells using RNA-seq technology

**DOI:** 10.1186/s40781-018-0177-5

**Published:** 2018-07-13

**Authors:** Shanti Choudhary, Wenli Li, Derek Bickhart, Ramneek Verma, R. S. Sethi, C. S. Mukhopadhyay, Ratan K. Choudhary

**Affiliations:** 1 School of Animal Biotechnology, Guru Angad Dev Veterinary and Animal Sciences University, Ludhiana, Punjab 101004 India; 2 Cell Wall Biology and Utilization Research, USDA-ARS, Madison, WI 53706 USA

**Keywords:** Goat, Milk fat globule, RNA sequencing, Xanthosine, RT-qPCR

## Abstract

**Background:**

Xanthosine treatment has been previously reported to increase mammary stem cell population and milk production in cattle and goats. However, the underlying molecular mechanisms associated with the increase in stem cell population and milk production remain unclear.

**Methods:**

Primiparous Beetal goats were assigned to the study. Five days post-partum, one mammary gland of each goat was infused with xanthosine (TRT) twice daily (2×) for 3 days consecutively, and the other gland served as a control (CON). Milk samples from the TRT and CON glands were collected on the 10th day after the last xanthosine infusion and the total RNA was isolated from milk fat globules (MEGs). Total RNA in MFGs was mainly derived from the milk epithelial cells (MECs) as evidenced by expression of milk synthesis genes. Significant differentially expressed genes (DEGs) were subjected to Gene Ontology (GO) terms using PANTHER and gene networks were generated using STRING db.

**Results:**

Preliminary analysis indicated that each individual goat responded to xanthosine treatment differently, with this trend being correlated with specific DEGs within the same animal’s mammary gland. Several pathways are impacted by these DEGs, including cell communication, cell proliferation and anti-microbials.

**Conclusions:**

This study provides valuable insights into transcriptomic changes in milk producing epithelial cells in response to xanthosine treatment. Further characterization of DEGs identified in this study is likely to delineate the molecular mechanisms of increased milk production and stem or progenitor cell population by the xanthosine treatment.

**Electronic supplementary material:**

The online version of this article (10.1186/s40781-018-0177-5) contains supplementary material, which is available to authorized users.

## Background

Purine nucleoside xanthosine (XS) when added to asymmetrically dividing stem cells, induceds a transition to symmetrical division, which ultimately leads proliferation of stem cells. Suppression of asymmetrical division of cells by XS is regulated through inosine monophosphate dehydrogenase (IMPDH) in p53 dependant fashion [1]. XS is converted into xanthosine 5′-monophosphate (XMP) by the enzyme IMPDH. Thus, XS has the essential role in IMPDH regulation. The intramammary infusion of xanthosine (XS) into lactating cow, has been shown to increase mammary stem cell population and has been hypothesized to increase milk production [2]. Contrarily, xanthosine treatment shown to have no effect on mammary stem cell population [3]. Therefore, to make a distinct and clear effects of XS on mammary gland, more research are warranted.

Milk is secreted by the mammary epithelial cells (MECs), which are gradually exfoliated during lactation. Because of the gradual exfoliation and constant replenishment of these MECs, it is likely that gene expression differences in these cells may be major contributors to the overall process of lactation. Several methodologies have been developed to isolate and enrich MECs from mammary glands for transcriptional profiling of MEC. One methodology involves the surgical biopsy of mammary tissue followed by the preparation of singular cell suspension and in vitro propagation of MECs in culture [4]. Alternatively, immunomagnetic methods of MEC cell seperatation from milk somatic cells have also been attempted by several studies [5, 6]. Recently, non-invasive approaches of extracting MECs from milk have been developed that involve the capture of milk fat globules (MFG) from the milk fat layer. These MFGs contain MEC cytoplasmic remnants, called crescents, due to their moon shaped appearance under the light microscopy [7]. The percentage of MFG crescents in milk varied among the mammalian species with a high percent volume (3–8%) in human milk to a low percent volume (< 1%) in cattle [8, 9]. MFG crescents, being its cytoplasmic origin, are a source of MEC-specific RNA. In the analysis of the human MFG transcriptome using microarrays and RNA-sequencing, it has been confirmed that the MFG transcriptome include genes uniquely expressed in the MEC [7, 10, 11]. These studies have established MFGs as a reliable source for MEC-specific gene expression analysis during lactation.

The main aim of the present study was to identify XS-induced, gene expression differences in MECs by deep sequencing of the total RNA associated with MFG during early lactation in Beetal goats. Using RNA-sequencing, we sought to identify the differentially expressed genes (DEGs) between XS treated (TRT) and control glands (CON). Additionally, using reverse transcriptase quantitative PCR (RT-qPCR), we sought to confirm observed differential gene expression and fully characterized the transcript abundance of genes of interest.

## Methods

### Animals and experimental design

Use of goats for this study was approved by the “Committee for the Purpose of Control and Supervision of Experiments on Animals” (CPCSEA; reference no. 25/20/2016), Ministry of Environment, Forest and Climate Change (Animal Welfare Division), New Delhi. Eight primiparous Beetal goats were used in this experiment. A 20 mL suspension of 10 mM XS was infused through the teat canal and 1 mL of intra-parenchymal injection was given to one randomly chosen mammary gland (half udder) for 3 consecutive days, twice daily (once in the morning, and once in the evening immediately after milking). Complete milk removal was performed for each TRT glands, followed by the insertion of a sterile 20 gauge blunt needle into the teat canal to extract all of the remaining milk secretion. The XS powder was diluted in sterile saline and sterilized with a 0.22 μm filter before infusions. To ensure the delivery of XS into the peripheral regions of the glands, an additional injection of 1 mL XS was administered intraparenchymally according to the methods used in a similar study [2]. For each goat, one mammary gland was treated with XS while the other gland was used as control (CON) and was not infused with XS. One week after the last XS injection, milk was collected for RNA isolation from both the TRT and CON glands of each animal.

### Milk image analysis

**Fig. 1:**
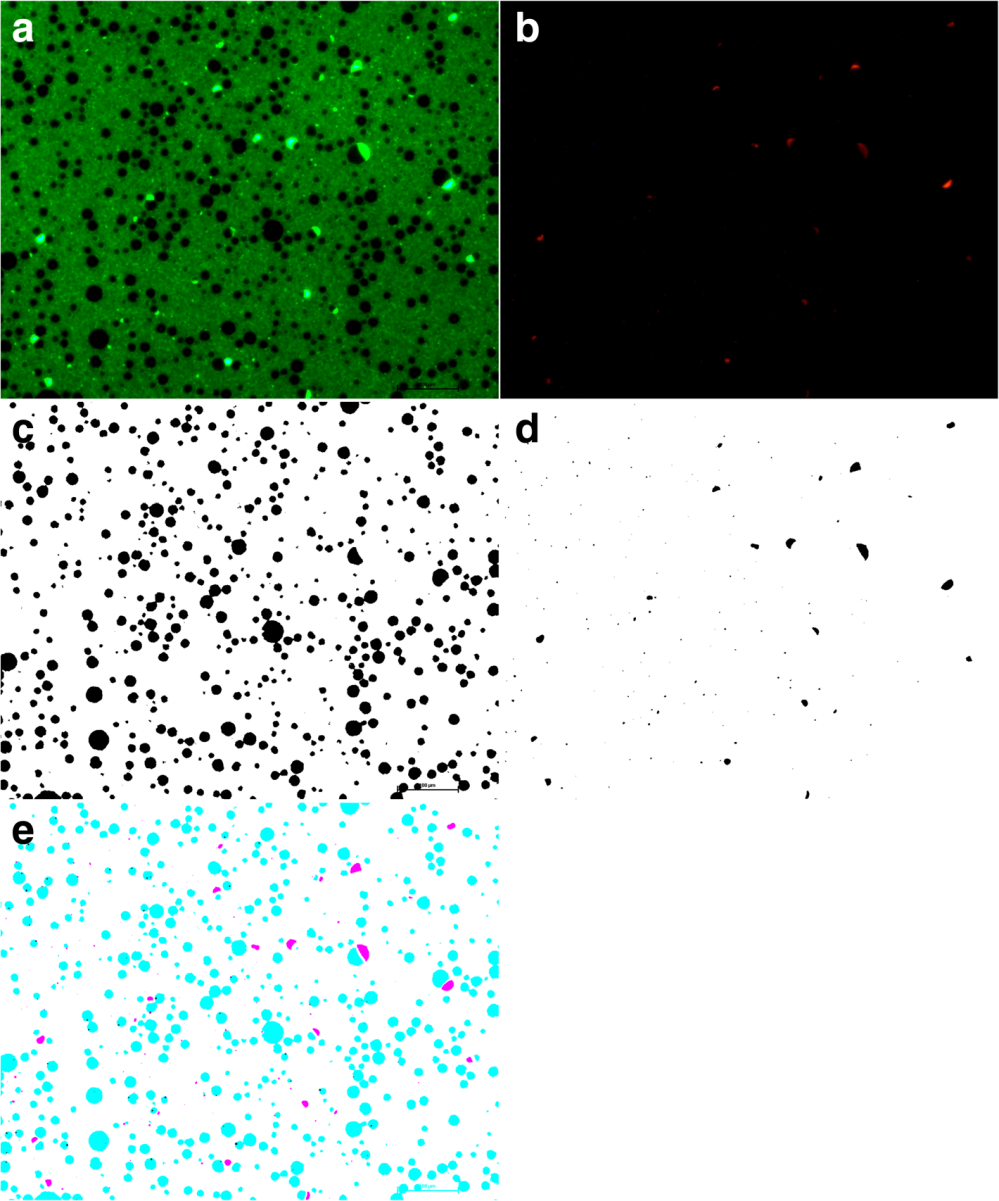
Image analysis of goat milk with acridine orange (AO) to visualize milk fat globules (MFG) and crescents associated with MFG. Each image contained two channels one for DNA (green) and the other for RNA (red). DNA appered green under FITC channel (**a**) wherease, RNA appered red under the TRITC channel (**b**). Dark sphereical like structures of MFG are evident. Grey images are extracted from both the channels (**c** and **c**, respectively) and merged together (**e**) to show the presence of crescents over MFG. These crescents and membrance over MFG were source of mammary epithelial cell specific RNA

Freshly collected milk were mixed with 0.1 0.1% acridine orange (AO) and incubated for 5 min at room temperature to stain nucleic acids (DNA and RNA). A 10 μL of AO-stained milk were placed onto positive charged slides and covered with a coverslip which was then sealed with nail polish to prevent milk evaporation. Slides were viewed immediately on an inverted fluorescent microscope (Nikon Eclipse 90i, Tokyo, Japan). Two images were captured for each slide, a red fluorescence channel TRITC for AO-intercalated RNA, and a green fluorescence channel FITC to view AO-intercalated DNA. AO-stained milk Images were analyzed using Fiji as described earlier [8]. The accuracy of this method was confirmed manually by comparing a subset of processed and original images. Overlays of the AO- RNA channel (Red) with the AO-DNA channel (green) images were analysed using Fiji [12] (Fig. 1).

### RNA isolation from milk fat globules

Total RNA was extracted using a combination of Trizol (Invitrogen, Carlsbad, CA, USA) and GenElute Mammalian Total RNA isolation kit (Sigma, St. Louis, MO, USA) as published earlier [13]. Briefly, for each 40 mL milk sample after centrifugation, 500–700 μg of milk fat was collected using a 1 mL sterile microtip, and put into a 5 mL nuclease free tube (Genaxy Scientific Pvt. Ltd. New Delhi, India) containing 1.5 mL of Trizol™ reagent (ThermoFisher Scientific, Waltham, USA). The milk fat was homogenized in Trizol for 2–3 min by vortexing. The homogenized mixture was incubated at room temperature (RT) for 3 min and stored at − 80 °C for further use. The homogenates were thawed quickly by rubbing with palm, vortexed briefly and centrifuged at 12000 x g for 5 min at 4 °C to remove excess fat (on top). Clean homogenate (mid layer) was transferred into a new, 2 mL RNAase-free tube, containing 300 μL of chloroform (HiMedia Laboratories Pvt. Ltd. Mumbai, India). After vigorous shaking for 30 s, the mixture was incubated at RT for 10 min for complete solubilization of fat. Tubes were centrifuged at 12000 x g for 15 min at 4 °C. A 300 μL of the clear supernatant was used for total RNA extraction using the GenElute Mammalian Total RNA isolation kit, following the manufacturer’s instructions. RNA quantity was measured using a NanoDrop 1000 (ThermoFiesher Scientific, Waltham, USA). RNA integrity was determined using a Tapestation (Agilent Technologies, Santa Clara, USA) before the samples were processed for RNA sequencing.

### Library preparation and RNA sequencing

Library preparation, quality control, sequencing and data analysis were performed at a commercial sequencing facilty (SciGenome, Kochi, India). Due to budget constraints and feasibility of RNA sequencing of milk fat derived RNA, only four libraries (two XS treated- 741 L and 647 L and two control- 741R and 647R) were considered from two individual goats. To generate each library, 5–10 μg of total RNA was isolated and prepared using a TruSeq RNA sample preparation kit V2 (Cat:RS-122-2001, Illumina, San Diego, CA, USA). Size distribution of the sequencing library was determined by gel electrophoresis. Library quantification was done using Qubit 3.0 fluorometric quantitation system (ThermoFisher Scientific, Waltham, USA). Paired-end sequencing was performed on Illumina’s HiSeq 2500 platform. Approximately 40 million paired-end, 2 × 100 bp reads were generated for each sample. Raw fastq files have been submitted to NCBI Sequence Read Archive (SRA) database with the study accession PRJNA389156 (https://www.ncbi.nlm.nih.gov/bioproject/PRJNA389156/).

### RNA-sequencing quality control, read mapping and data analysis

Initial checking of raw sequences for the quality has been performed by the sequencing vendor. Briefly, reads containing adaptors were filtered from the dataset. To avoid base composition bias, 12 bases were trimmed at the 5′ end of both read pairs. RNA sequencing read quality checks included base quality score distribution, sequence quality score distribution, average base content per read, GC distribution in the read and occurrence of over-represented sequences. Raw reads were filtered when the average of their base quality scores was less than 20. Processed reads were aligned to the goat reference genome downloaded from NCBI (GCA_001704415.1_ARS1) [14]. Sequencing reads from non-coding RNAs were excluded from the analysis. Sequence alignment was performed using the STAR program (ver.2.5.2b). Two methods were used for differential gene expression analysis. First, the quantification of the raw expression of genes and transcripts was performed using the cufflinks program (ver. 2.2.1). The expression values were normalized to a FPKM (fragment per kilo per million) value for each of the genes and transcripts. Differential expression analysis was performed using cuffdiff program within the cufflinks package. Second, Deseq2 [15] was also used to identify significant differentially expressed genes. In this analysis, RUVseq [16] was used to remove any potential unwanted variation in gene quantification using several housekeeping genes. We compared the mRNA profile of TRT samples to that of the CON samples. A total of four mRNA sequence datasets (741 L, 741R, 647 L and 647R, as described in previous section) were used.

### cDNA synthesis and RT-qPCR

First-strand cDNA was synthesized from 1000 ng of total RNA using iScript Reverse Transcription Supermix (BioRad Laboratories, CA, USA) according to the manufacturer’s instructions. Resultant cDNA was stored at − 20 °C. The RNA isolated from MFG was used to quantify changes in gene expression by real time thermocycler (CFX96 Touch™ Real-Time PCR) using iTaq™ Universal SYBR Green Supermix (BioRad Lab. California, USA). Gene specific primers were designed on the basis of goat reference mRNA sequence obtained from NCBI using NCBI Primer-BLAST following guidelines described previously [17]. Desalted oligos (25 mM) were purchased from Integrated DNA Technologies (IDT Inc., Iowa, USA).

Each 10 μL of PCR reaction contained each 0.25 μL of forward and reverse primers (2.5 pmol), 5 μL of Brilliant III Ultra-Fast SYBR® QPCR (Agilent Technologies, Santa Clara, USA), 3 μL of cDNA (1:10 dilution) and 1.5 μL of nuclease free water. Two-step RT-qPCR assays were performed with the following cycling conditions: 95 **°**C for 3 min; 340 repeating cycles of 95 °C for 10 s (denaturation) at respective annealing temperature (Additional file 1: Table S1) for a set of primers for 30 s (annealing); 95.0 **°**C for 3 min, 65.0 **°**C for 30 s, and then a temperature increase at 0.5 **°**C increment to 95.0 **°**C (melting curve). Each assay included a no-template control and a no reverse-transcriptase control. For no template control, 1 μL of RNase/DNase free water was used as template. For the no reverse-transcriptase control, reverse-transcriptase enzyme was excluded during cDNA synthesis and resultant cDNA used as template.

A total set of 22 genes were selected for RT-qPCR analysis. Genes that are expressed in MEC during lactation and affected by the XS treatment, were selected as candidate genes for RT-qPCR analysis. A subset of DEGs obtained from RNA-seq data were also selected to validate the sequencing data. The *RPL4* and *RPS23* were selected as reference genes as reported in earlier study [18]. Threshold (Ct) values of target genes were normalized to the geometric mean of two reference genes to obtain ΔCt values (target gene Ct – reference gene mean Ct = ΔCt) [19]. All reactions were conducted in duplicate and Ct values were averaged for each tested gene-condition-sample combination.

### Statistical analysis

For the functional annotation and pathway analyses of RNA-seq data, pathways were tested for statistical significance using a false positive threshold of 0.05 after Bonferroni’s correction. For the RT-qPCR data analysis, the raw ΔCt values of genes were log_2_ transformed after discovering that their values were not normally distributed (estimated by Shapiro Wilk’s Test; data not shown). Paired t-tests were employed to test significant differences among gene-condition comparisons. Data were analysed using the SPSS ver. 22 (IBM SPSS Statistics for Windows Armonk, NY). A *p*-value < 0.05 were considered statistically significant value.

## Results

### Total RNA extraction from milk fat

We obtained a high quality of total RNAs from goat milk fat. The total RNA concentration averaged 770.12 ± 754.7 ng/μl (mean ± SD). RNA extraction protocol provided high purity for extracted RNA having optical density (OD) ratios of 260/280 and 260/230 to 2.04 ± 0.04 and 1.84 ± 0.38 (mean ± SD), respectively. All the RNA samples of goat milk fat were suitable for RT-qPCR analysis. Samples having RNA integrity numbers (RIN) greater than 7 were sent for RNA sequencing. RIN of other RNA samples were between 6 and 7 and were suitable for RT-qPCR analysis.

### RNA-seq and mammary transcriptome

Four RNA samples (RIN > 7.0) were selected for RNA sequencing from an initial group of 16 samples. The percentage of mapped reads to the ARS1 goat reference assembly [14] averaged 93.4 + 2.1 (Mean ± SD) for each sequenced sample. There were 10,267 to 11,470 genes per sample with an FPKM value ≥ 0.2, indicating some level of detectable transcription (Table 1). Functional annotation of these transcripts were enriched for cellular and metabolic pathways that were involved with catalytic and binding activities (Additional file 1: Table S2). In each of the four RNA sequencing samples, majority of the transcripts were lowly expressed (FPKM < 15; 84.3%). One animal (741) had fewer number of DEG than the other tested sample (647). This discrepancy could have been influenced by the difference in animal genetics, or due to the differences in mammary gland permeability that allowed xanthosine to diffuse into the CON gland. For abundantly expressed genes (FPKM ≥ 500), we observed an enrichment of cellular and metabolic processes (on the basis of PANTHER classification system). Transcripts corresponding to milk protein genes (*CSN3, LALBA, PAEP, GLYCAM1, TPT1, PLIN2* and *FABP3*) were the most abundantly expressed genes. Our study showed high expression of perilipin 2 (*PLIN2*- a protein involved in milk fat globule formation), fatty acid binding protein (*FABP3*), and many ribosomal proteins during early lactation in goats. The *PLIN2* gene abundantly expressed in macaque milk (Lemay et al., 2013) and *FABP3* gene in cattle [20].

**Table 1:** Whole transcriptomic signature (FPKM ≥ 0.2) of milk fat globules RNA expressed in xanthosine treated (TRT) and control glands (CON) during early lactation of Beetal goats

# of genes	647 L (TRT)	647R (CON)	741 L (TRT)	741R (CON)	Mean of all four samples
Abundantly expressed	112	110	115	111	112 (1%)
Moderately expressed	171	203	186	178	184.5 (1.7%)
Rarely expressed	1253	1564	1402	1402	1405 (12.9%)
Very rarely expressed	8731	9593	9171	9162	9164 (84.3%)
Total number of genes	10,267	11,470	10,874	10,853	10,866
Difference in # of genes between TRT and CON glands	1203		21		

### Functional annotation analysis of abundantly expressed genes

**Fig. 2:**
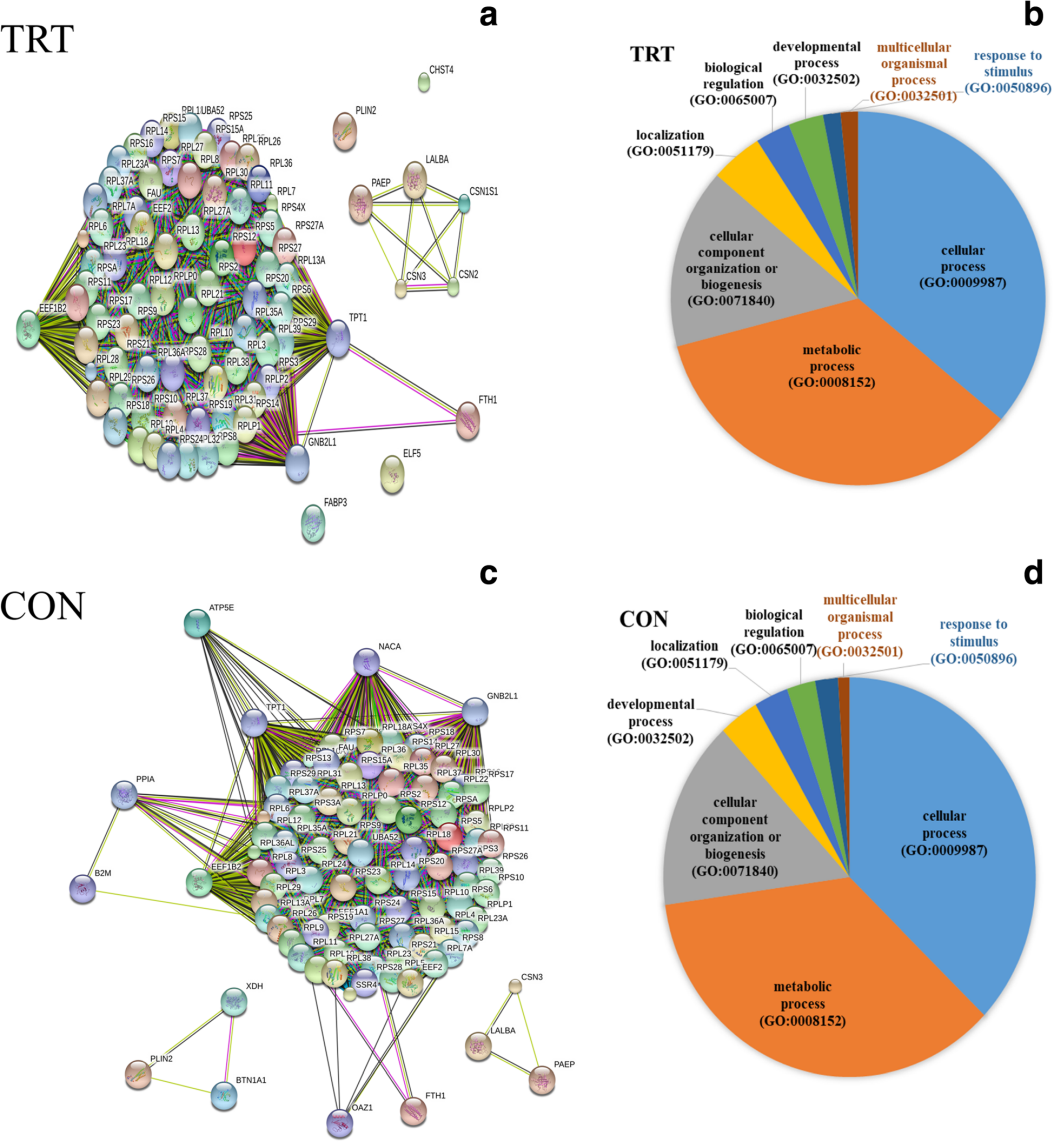
Categorization of abundantly expressed genes based on GO:Biological Process**.** Abundantly expressed genes of TRT group showing strong hub of ribosomal pathway (**a**) and their Molecular Function (**b**). Likewise, in CON group ribosomal pathway (**c**) and majority of these genes were involved with cellular and metabolic process (**d**) similar to TRT group

To characterize functions of most abundantly expressed genes (FPKM ≥500), list of genes were subjected to gene ontology (GO) classification and functional analysis using PANTHER [21]. In the TRT group, three key genes, *GNB2L1*, *EEF1B2* and *TPT1,* encode key ribosome-associated proteins **(**Fig. 2a). Consistently, KEGG pathway analysis showed significant enrichment of ribosome-associated gene families, including the structural constituents of ribosomal genes (*RPL4, RPS9, RPS10* and other ribosomal genes). Our data indicated that the highly expressed genes are mainly involved in cellular process (GO:0009987), metabolic process (GO:0008152) and cellular component organization (GO:0071840) (Fig. 2b). Additionally, milk protein genes (*LALBA, CSN2, CSN3, PAEP, CSN1S1*) were highly expressed in both the TRT and CON groups.

### Functional annotation of differentially expressed genes (DEGs)

A total of 58 genes were differentially expressed MEC after XS treatment of mammary glands. A majority of DEGs (46) were found to be down-regulated after XS treatment whereas 12 genes were up-regulated (Table 2). Pathway analysis of DEG showed enrichment of genes for the following biological processes: defense response to other organsm, cytokine regulated signalling pathway, regulation of the response to other stimuli, regulation of cytokine production, and response to endogenous stimulus. The most prevalent GO-CC (cellular component) terms were associated with the cell surface (10 genes) and the plasma membrane (15 genes). Among the 10 up-regulated genes, both *B2M* and *LTF* are of particular interest. Our data did not show the up-regulation of heat shock protein genes in the TRT glands, suggesting that XS infusion did not cause mammary tissue damage.

**Table 2:** List of differentially expressed genes (DEGs) of goat mammary epithelial cells after xanthosine infusion into the gland

Genes	log2 Fold Change	*p*-value	Name of protein
1. CXCR2	−1.017323	3.11E-05	C-X-C Motif Chemokine Receptor 2
2. SAMD9	−0.8044989	0.00048	Sterile alpha motif domain-containing protein 9
3. LOC102168687	−0.798857	0.00067757	interferon-induced protein with tetratricopeptide repeats 1
4. FOS	−0.7106714	0.00150655	Proto-oncogene c-Fos
5. LOC106502722	−0.706349	0.00609026	elongation factor 1-alpha 1 pseudogene
6. CXCR1	−0.7019749	0.00156437	C-X-C Motif Chemokine Receptor 1
7. MXD1	−0.6870472	0.00377718	Max dimerization protein 1
8. TNFRSF1B	−0.6679126	0.00536264	Tumor necrosis factor receptor superfamily member 1B precursor
9. CLEC5A	−0.6519524	0.00334639	C-type lectin domain family 5 member A
10. ANTXR2	−0.6468424	0.00671523	Anthrax toxin receptor 2 precursor
11. EMB	−0.6415234	0.00454368	Embigin
12. LOC102180659	−0.6380424	0.01443435	elongation factor 1-alpha 1 pseudogene
13. PLEK	−0.6376403	0.00658301	Pleckstrin
14. DUSP1	−0.6345636	0.00825818	Dual specificity protein phosphatase 1
15. IFIT1	−0.6331932	0.00311884	subfamily A member 5
16. PECAM1	−0.6115827	0.00567366	Platelet endothelial cell adhesion molecule
17. FAM65B	−0.6020531	0.01551968	Protein FAM65B
18. ISG15	−0.591961	0.00810711	Ubiquitin-like protein ISG15
19. NCF2	−0.5901478	0.00763365	Neutrophil cytosol factor 2
20. C5AR2	−0.5877041	0.00674769	Complement Component 5a Receptor 2
21. SAMSN1	−0.5803822	0.0086767	SAM domain-containing protein SAMSN-1
22. MX1	−0.5801172	0.01102704	Interferon-induced GTP-binding protein Mx1
23. PTAFR	−0.5571907	0.00830068	Platelet-activating factor
24. VSIR	−0.546773	0.02426786	V-Set Immunoregulatory Receptor
25. JUNB	−0.5417107	0.0151331	Transcription factor jun-B
26. PLAU	−0.5322312	0.00924439	Urokinase-type plasminogen activator
27. RSAD2	−0.5225815	0.01513903	Radical S-adenosyl methionine domain-containing protein 2
28. DUSP2	−0.5221989	0.01338515	Dual specificity protein phosphatase 2
29. HCLS1	−0.5198929	0.00978145	Hematopoietic lineage cell-specific protein
30. SSH2	−0.519027	0.01453977	Slingshot Protein Phosphatase 2
31. SYNE1	−0.5130198	0.04286633	Spectrin Repeat Containing Nuclear Envelope Protein 1
32. NOTCH1	−0.5090343	0.01658702	Notch 1 protein receptor
33. CYTIP	−0.5084053	0.01266586	Cytohesin-interacting protein
34. SELL	−0.4994215	0.01581525	L-selectin precursor
35. THBS1	−0.4897332	0.02190149	Thrombospondin-1 precursor
36. LDH-A	−0.4831976	0.06139028	Lactate Dehydrogenase A
37. PSTPIP2	−0.4826517	0.02163436	Proline-serine-threonine phosphatase-interacting protein 2
38. ARHGAP45	−0.4824378	0.02094921	Rho GTPase Activating Protein 45
39. HCAR2	−0.4787442	0.03908657	Hydroxycarboxylic Acid Receptor 2
40. HCK	−0.4786929	0.0215455	Tyrosine-protein kinase HCK isoform 2
41. ADGRE1	−0.4760767	0.01657563	Adhesion G Protein-Coupled Receptor E1
42. HK3	−0.4725703	0.0154585	Hexokinase-3
43. RGS2	−0.4722578	0.05622303	Regulator of G-protein signaling 2
44. RGS14	−0.4673717	0.02127159	Regulator of G-protein signaling 14
45. TMEM154	−0.4579886	0.07281125	Transmembrane Protein 154
46. C5AR1	−0.4556632	0.02037596	Complement C5a Receptor 1
47. MBLAC2	0.40364221	0.12046919	Metallo-beta-lactamase domain-containing protein 2
48. B2M	0.4105621	0.09027626	beta-2-microglobulin
49. LOC108635405	0.4289959	0.08146865	collagen alpha-1(II) chain-like
50. TAF15	0.43142113	0.0956541	TATA-Box Binding Protein Associated Factor 15
51. COX7A2	0.46584597	0.07022808	cytochrome c oxidase subunit 7A2
52. LOC108635296	0.50689456	0.03272288	collagen alpha-1(I) chain-like
53. LTF	0.54549056	0.03459662	Lactotransferrin Lactoferricin-B
54. LOC108635078	0.55728309	0.0257517	basic proline-rich protein-like
55. RPL37A	0.60565486	0.01970951	putative 60S ribosomal protein L37a
56. LOC108634776	0.6232389	0.00864377	zinc finger protein 532-like
57. LOC108634774	0.70202338	0.00454456	collagen alpha-1(I) chain-like
58. LOC108635079	0.71456286	0.00591021	basic salivary proline-rich protein 1-like

**Fig. 3:**
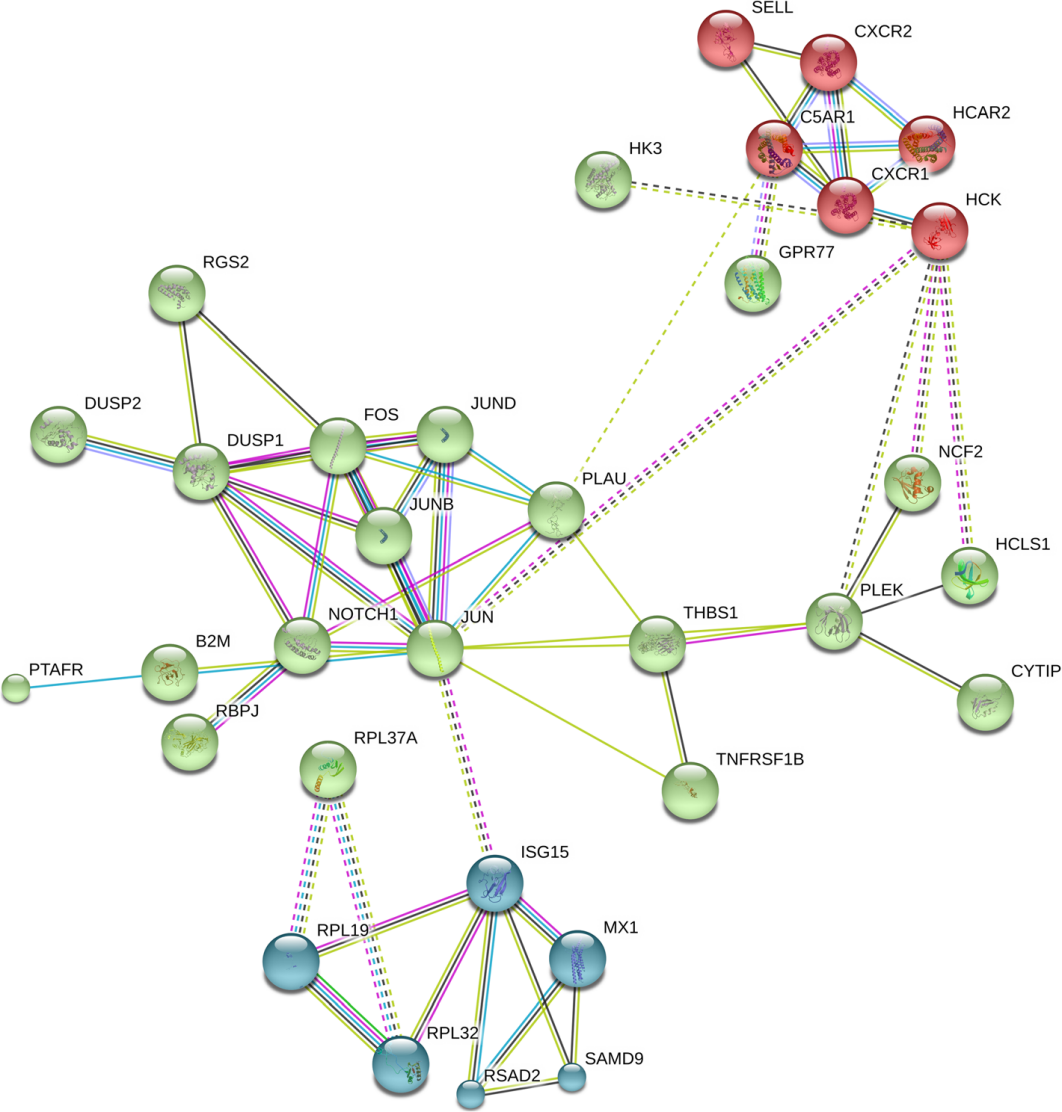
Visualization of protein interaction neworks of diffentially expressed genes between the TRT and CON group. Network showing 3 k-means clustering indicated by three different colors and their interactions

PANTHER pathway enrichment analyses showed down-regulation of inflammation signaling pathway mediated by chemokine and cytokine (CXCR1, CXCR2, *PTAFR, RGS14, C5AR* and *JUNB*) in TRT group. Notably, adhesion molecules *(PECAM1, SSH2, SYNE1, CYTIP, SELL*) were down-regulated upon XS treatment. Next, we investigated protein-protein interactions of DEGs using the STRING database. Out of 58 DEGs, STRING identified only 47 human homologs for calculating protein-protein interaction with a predicted confidence score of > 0.4. The interaction enrichment analysis result showed that the network has significantly more interactions (PPI enrichment *p*-value 2.3e-09) than the expected and the entire network contained three sub-divisions (Fig. 3) indicative of three distinct biological processes.

### RT-qPCR confirmation of genes of interest

An elaborate RT-qPCR analyses of many target genes were done for three explicit purposes: 1) to validate RNAseq data; 2) to assess whether MFG RNA are quantitatively representative of the transcriptional information contained in MEC; and 3) to assess the potential effect of XS on milk production. Major milk protein genes (*LALBA* and *CSN2*), steroid receptors (*ESR1* and *PRB*), proliferation (*PCNA*) and apoptosis marker (*TP53*) and cell differentiation marker (*MUC1*) were analysed in addition to DEGs.

**Fig. 4:**
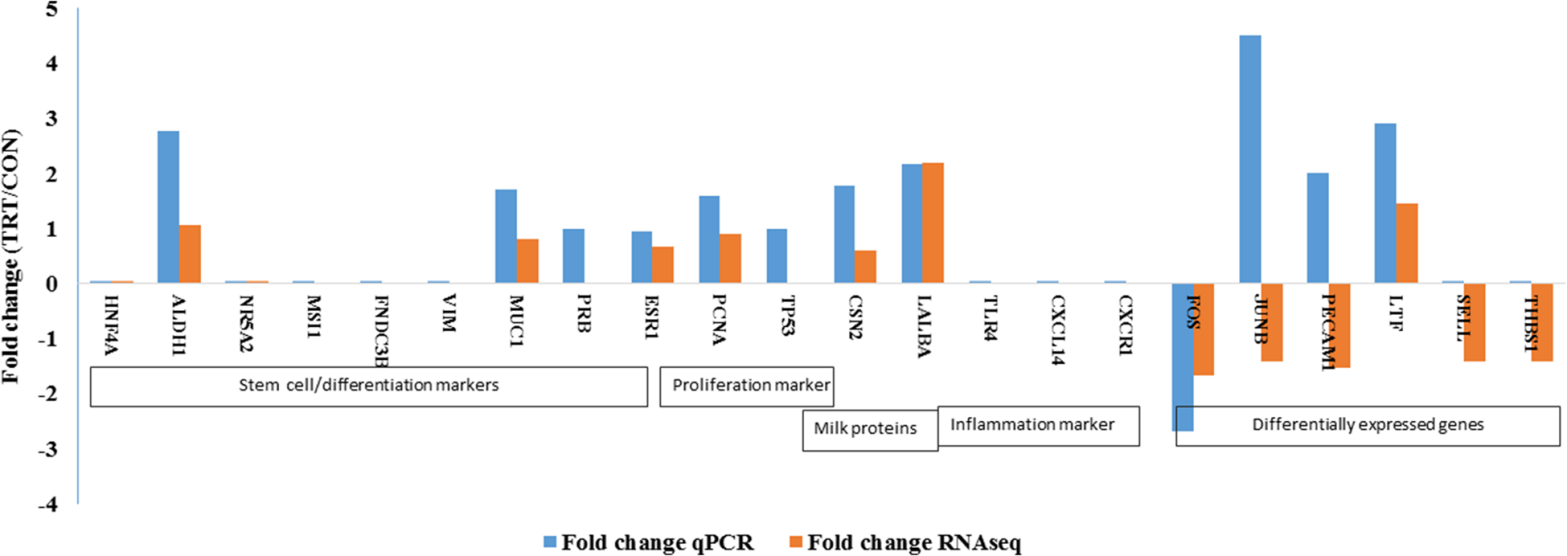
Validation of genes of interest by RT-qPCR. Genes which were abundantly expressed and did not expressed in RNAseq data were validated. Furthermore, differentially expressed genes of RNA-seq date were analyzed to validate RNA-seq data

Our RT-qPCR experiments validated many of our findings. We confirmed an absence of expression of stem cell markers (*HNF4A NR5A2, MSI1, FNDC3B*) that were not present in RNA-seq data. Among the list of DEGs, lactotransferrin (*LTF*) was up-regulated whereas, Fos proto-oncogenes (*FOS*) was down-regulated in TRT. RT-qPCR analyses showed similar pattern of expression as observed in DEG of RNA-seq data. However, our qPCR result was not consistent with the RNA-seq expression results of *JUNB* and *PECAM1* genes*.* This difference may be attributed because of low abundance of transcripts that limits the detection by qPCR. We also failed to amplify two genes*, SELL* and *THBS1,* using RT-qPCR (Fig. 4). Later, we confirmed the extremely low abundance of *SELL* and *THBS1* gene transcripts in milk fat RNA using droplet digital PCR (data not shown).

## Discussion

The effects of XS treatment in the culture of bovine mammary epithelial cells have been shown, in vitro*,* to increase cell population and enhance symmetric cell division [22]. A non-invasive, in vivo method is desirable for understanding the role of XS treatment in promoting mammary epithelial cell proliferation, as an increase in the population of such cell is directly associated with milk producing ability of the glands [23]. A milk-based assay could be used to track gene expression of milk producing mammary epithelial cells [11]. It has been well documented by many researchers that the milk fat fraction is rich in RNA transcripts specific to mammary epithelial cells, as these are devoid of stromal cells, nerve cells, endotheial cells, fat cells and inflammatory cells [24]. Though the number of cytoplasmic crescents in ruminant milk is fewer in count than it is in human and macaque milk, the sensitivity of our RNA-seq analysis of milk fat isolates meant that our study was not limited by the lower proportion of cytoplasmic crescents in goat milk [25]. We demonstrate that this method provides abundant quantity and good quality of RNA suitable for RNA sequencing. This shows consistency with the findings of other studies, in which milk fat has been used to assess the transcriptome profile of milk production related genes in human [7], buffalo [26], bovine [27] and goat [28].

An advantage of our study is that XS treated (TRT) and control (CON) glands were present with the same goat. This should reduce any biological bias resulting from the sampling of different individual goats due to different genetic profiles. Thus, our experimental method captured true variation in milk producing cells affected by XS treatment, with less noise due to a different genetic background of sampled individuals. However, we cannot entirely rule out genetics as a major determinant of XS treatment response as our data also indicated that there is an individual-specific responses. This finding should be considered and evaluated for the future application of XS treatment in goats and other dairy animals for milk production.

Several genes, *LALBA* (α-lactalbumin), *CSN2* (β-casein), *CSN1S1* (α-S1-casein), CSN3 (κ-casein), *GLYCAM1* (glycosylation dependent cell adhesion molecule-1) and *CSN1S2* (casein-α-S2), were abundantly expressed in sheep mammary gland during lactation [29]. In our study, these genes are among the highest expressed genes found in our milk fat samples during early lactation. Additionally, we observed abundant expression of tumor protein translationally-controlled 1 (*TPT1*), fatty acid binding protein 3 (*FABP3*), perilipin 2 (*PLIN2*) and ribosomal proteins, which were among the top 20 abundantly expressed genes of goat mammary gland (Additional file 1: Table S3). FABP3 protein is a candidate for tumor suppressor of human breast cancer with a suggested role in arresting growth of mammary epithelial cells. *TPT1* has been shown to reduce oxidative stress, minimize apotosis and promote cell survival in a p53-dependant manner [29]. *PLIN2* is associated with MFG membrane and found in a wide variety of cells, including lactating mammary epithelial cells. Additionally, *PLIN2* regulates lipid and protein metabolism in lactating dairy goats [30]. It is suggested that elevated expression of *TPT1, PLIN2* and *FABP3* might play a role in suppressing goat mammary tumor formation. Precisely, the role of *FABP3* has been associated to import/export of fatty acids in the cell and highly upregulated during bovine lactation [31]. Although, reports on the incidence of mammary tumors in ruminants are rare [32], our group has encountered epithelial-mesenchymal transition, mammary epithelial cell hyperplasia and mammary pre-cancer in both goats and water buffalo [33, 34] with no sign of mammary tissue damage.

We found that XS administration twice a day for 3 days consecutively, altered the gene expression in TRT glands in goats compared to an non-inoculated CON gland on the same animal. Differential response of XS treatment was evidenced by varied number of DEGs between TRT and CON glands. The duration of XS administration (3 days) was based on an inosine study conducted in transgenic goat [35]. XS did prouduce deleterioius effects to the gland neigther causes any inflammatory reactions. We observed absence of mesenchymal cell marker (*VIM*), and inflammatory markers (*TLR4, CXCL14* and *CXCR1*) amplification in the RNA harvested from mlk fat layer, indicating XS did not cause MEC inflammation. A pronounced effects of XS treatment on goat milk production may require further optimization of XS dose and duration, and may need to account for animal genetic variation. It is also imperative to note that a comparison of effects of inosine and xanthosine should be tested individually to evaluate if inosine administration is more suitable for enhancing milk production. In using Beetal goats as a test subject, the disparity of left and right mammary half gland should be considered for evaluation of true milk production potentials. The down-regulation of a dominant portion (48 out 58) of the identified DEGs, like *FOS, PECAM1, JUNB, SELL, THBS1*, indicated XS primarily down-regulates the expresson of genes. Platelet and endothelial cell adhesion molecule (PECAM1), selectin L (SELL) and Thrombospondin 1 (THBS1) are the cell surface adhesion molecules that mediates cell-to-cell and cell-to-matrix intereactions. Down-regulation of cell adhesion molecules is typically associated with an inhibition of cell growth [36]. Due to the limited number of goats used in this study, the number of DEG was limited. We did not find significant enrichment of KEGG pathways from the list of DEG; however, we did find several Gene Ontology (GO) terms for biological process were enriched for immune responses and cellular response to organic substance.

Previous studies have showed a pronounced effect of inosine on milk production in transgenic goats [35] suggesting that exogenous nucleosides may stimulate milk production in ruminant species. PANTHER pathway analysis revealed that several XS-induced down-regulated genes were involved in inflammation signalling pathway, which is mediated by chemokine and cytokine detection by the cells. When activated, this pathway could promote chemokine-induced adhesion and migration of leukocytes in the local tissue. We hypothesize that the down regulation of inflammatory signaling pathways induced by XS, could prevent the excessive recruitment of leukocytes to inflammatory sites. XS up-regulated expression of anti-bacterial genes (*LTF* and *B2M*), which may be beneficial to the host by preventing the incidence of mastitis. The Beta-2-microglobulin (*B2M*) gene codes for a protein that is associated with the major histocompatibility complex class 1 (MHC I) and is a precursor of an anti-bacterial chemokine [37]. Lactotransferrin (*LTF*) is a gene that encodes a major iron binding milk protein. *LTF* has a wide spectrum of properties that include anti-bacterial, anti-viral, and anti-cancer activities and is involved in the regulation of cellular growth [38]. Future experiments are warranted to further investigate whether XS inoculation may increase general mammary gland health in ruminants.

In our candidate gene expression analysis using RT-qPCR, we found increased expression of ALDH1A1, a mammary stem marker [39], in the TRT. We were unable to amplify two DEGs namely, SELL and THBS1 in RT-qPCR. One possibility for the inconsistency could be the low amount of transcripts in our cDNA, evidenced by Ct value of these target genes > 35 cycles. In comparison to RNA-seq, RT-qPCR has less sensitivity in accurately detecting low abundance transcripts. Droplet digital PCR (ddPCR) is useful for low abundance targets and is better than the qPCR [40]. This observation supports the facts that mammary stem cells and progenitor cells may be present in the milk [41, 42]. However, we failed to quantify the transcripts of other putative mammary stem cell markers. Absence of basal or mesenchymal cell marker gene (vimentin or *VIM*) expression in our study indicated that RNA extracted from milk fat is specific to luminal mammary epithelial cells but not the basal or myopethelial cells. *VIM* is a basal or myoepithelal cell marker. Absence of expression of inflammatory markers (*TLR4, CXCR1, CXCL14*) provided the evidence that RNA harvested from milk fat is mainly derived from epithelial cells but not from the polymorphonuclear cells which was consistent with previous findings [27].

## Conclusions

This study characterized the global gene expression profile of goat mammary epithelial cells obtained form milk fat layer and characterized XS inoculation-associated differential gene expression in mammary epithelial cells of early lactating Beetal goat. Our study showed individual goats responded to XS treatment differently, suggesting a genetic predisposition to the treatment persists. Our findings further indicated that the administration of XS into the mammary gland has roles in down-regulation of inflammation signalling pathway, cell adhesion molecules and up-regulation of anti-bacterial genes. Down-regulation of inflammation signal and up-regulation of anti-bacterial genes may provide beneficial effects to mammary gland health. Our findings are promising. However, they were limited by a small number of goats (*n* = 2) included in this study, indicating that future studies with more number of animals are warranteed.

## Additional file

Additional file 1: **Table S1.** List of RT-qPCR primers sequences, product length and their annealing temperatures. **Table S2.** GO terms (Biological Process, Cellular Components and Molecular Functions) of all expressed genes of goat mammary epithelial cells during early lactation. **Table S3.** Top 20 transcripts identified by RNA-seq in goat mammary epithelial cells harvested from milk fat layers during early lactation in goat. (DOCX 29 kb)

## Abbreviations

CON: control
DEGs: differentially expressed genes.
MEC: mammary epithelial cells
MFG: milk fat globules
RT-qPCR: reverse transcriptase quantitative PCR
TRT: treatment
XS: xanthosine

## References

[1] Rambhatla L, Ram-Mohan S, Cheng JJ, Sherley JL (2005). Immortal DNA strand cosegregation requires p53/IMPDH-dependent asymmetric self-renewal associated with adult stem cells. Cancer Res.

[2] Capuco AV, Evock-Clover CM, Minuti A, Wood DL (2009). In vivo expansion of the mammary stem/ progenitor cell population by xanthosine infusion. Exp Biol Med (Maywood).

[3] Rauner G, Barash I (2014). Xanthosine administration does not affect the proportion of epithelial stem cells in bovine mammary tissue, but has a latent negative effect on cell proliferation. Exp Cell Res.

[4] Prpar Mihevc S, Ogorevc J, Dovc P (2014). Lineage-specific markers of goat mammary cells in primary culture. Vitr Cell Dev Biol Anim.

[5] Boutinaud M, Jammes H (2002). Potential uses of milk epithelial cells: a review. Reprod Nutr Dev.

[6] Anand V, Dogra N, Singh S, Kumar SN, Jena MK, Malakar D (2012). Establishment and characterization of a buffalo (Bubalus bubalis) mammary epithelial cell line. PLoS One.

[7] Lemay DG, Ballard OA, Hughes MA, Morrow AL, Horseman ND, Nommsen-Rivers LA (2013). RNA sequencing of the human milk fat layer transcriptome reveals distinct gene expression profiles at three stages of lactation. PLoS One.

[8] Choudhary RK, Kaur H, Choudhary S, Verma R (2015). Distribution and analysis of milk fat globule and crescent in murrah buffalo and crossbred cow. Proc Natl Acad Sci India Sect B Biol Sci.

[9] Ménard O, Ahmad S, Rousseau F, Briard-Bion V, Gaucheron F, Lopez C (2010). Buffalo vs. cow milk fat globules: size distribution, zeta-potential, compositions in total fatty acids and in polar lipids from the milk fat globule membrane. Food Chem.

[10] Maningat PD, Sen P, Rijnkels M, Sunehag AL, Hadsell DL, Bray M (2009). Gene expression in the human mammary epithelium during lactation: the milk fat globule transcriptome. Physiol Genomics.

[11] Lemay DG, Hovey RC, Hartono SR, Hinde K, Smilowitz JT, Ventimiglia F, et al. Sequencing the transcriptome of milk production: milk trumps mammary tissue. BMC Genomics 2013;14:872. 10.1186/1471-2164-14-872 PMC3871720 24330573

[12] Schindelin J, Arganda-Carreras I, Frise E, Kaynig V, Longair M, Pietzsch T (2012). Fiji: an open-source platform for biological-image analysis. Nat Methods.

[13] Choudhary S, Choudhary RK. Rapid and efficient method of total RNA isolation from milk fat for transcriptome analysis of mammary gland. Proc Natl Acad Sci India Sect B Biol Sci. 2017;

[14] Bickhart DM, Rosen BD, Koren S, Sayre BL, Hastie AR, Chan S (2017). Single-molecule sequencing and chromatin conformation capture enable de novo reference assembly of the domestic goat genome. Nat Genet.

[15] Love MI, Huber W, Anders S (2014). Moderated estimation of fold change and dispersion for RNA-seq data with DESeq2. Genome Biol.

[16] Risso D, Ngai J, Speed TP, Dudoit S (2014). Normalization of RNA-seq data using factor analysis of control genes or samples. Nat Biotechnol.

[17] Ye J, Coulouris G, Zaretskaya I, Cutcutache I, Rozen S, Madden TL (2012). Primer-BLAST: a tool to design target-specific primers for polymerase chain reaction. BMC Bioinformatics.

[18] Kapila N, Kishore A, Sodhi M, Sharma A, Kumar P, Mohanty a K (2013). Identification of appropriate reference genes for qRT-PCR analysis of heat-stressed mammary epithelial cells in riverine buffaloes (Bubalus bubalis). ISRN Biotechnol.

[19] Vandesompele J, De Preter K, Pattyn F, Poppe B, Van Roy N, De Paepe A (2002). Accurate normalization of real-time quantitative RT-PCR data by geometric averaging of multiple internal control genes. Genome Biol.

[20] Spitsberg VL, Matitashvili E, Gorewit RC (1995). Association and coexpression of fatty-acid-binding protein and glycoprotein CD36 in the bovine mammary gland. Eur J Biochem.

[21] Mi H, Huang X, Muruganujan A, Tang H, Mills C, Kang D, et al. PANTHER version 11: expanded annotation data from gene ontology and Reactome pathways and data analysis tool enhancements, Nucleic Acids Res 2017;45:D183–D189. 10.1093/nar/gkw1138 PMC5210595 27899595

[22] Choudhary RK, Capuco AV (2012). In vitro expansion of the mammary stem/progenitor cell population by xanthosine treatment. BMC Cell Biol.

[23] Akers RM, Capuco AV, Keys JE (2006). Mammary histology and alveolar cell differentiation during late gestation and early lactation in mammary tissue of beef and dairy heifers. Livest Sci.

[24] Zhang X, Liu N, Ma D, Liu L, Jiang L, Zhou Y (2016). Receptor for activated C kinase 1 (RACK1) promotes the progression of OSCC via the AKT/mTOR pathway. Int J Oncol.

[25] Choudhary S, Choudhary RK. Rapid and efficient method of total RNA isolation from milk fat for transcriptome analysis of mammary gland. Am Dairy Sci Assoc Annu Meet, J Dairy Sci. 2017;100(Suppl. 2)

[26] Chen Q, Wu Y, Zhang M, Xu W, Guo X, Yan X (2016). Milk fat globule is an alternative to mammary epithelial cells for gene expression analysis in buffalo. J Dairy Res.

[27] Cánovas A, Rincón G, Bevilacqua C, Islas-Trejo A, Brenaut P, Hovey RC (2014). Comparison of five different RNA sources to examine the lactating bovine mammary gland transcriptome using RNA-sequencing. Sci Rep.

[28] Brenaut P, Bangera R, Bevilacqua C, Rebours E, Cebo C, Martin P (2012). Validation of RNA isolated from milk fat globules to profile mammary epithelial cell expression during lactation and transcriptional response to a bacterial infection. J Dairy Sci Elsevier.

[29] Paten AM, Duncan EJ, Pain SJ, Peterson SW, Kenyon PR, Blair HT (2015). Functional development of the adult ovine mammary gland--insights from gene expression profiling. BMC Genomics.

[30] Shi H, Zhu J, Luo J, Cao W, Shi H, Yao D (2015). Genes regulating lipid and protein metabolism are highly expressed in mammary gland of lactating dairy goats. Funct Integr Genomics.

[31] Bionaz M, Loor JJ (2008). ACSL1, AGPAT6, FABP3, LPIN1, and SLC27A6 are the most abundant isoforms in bovine mammary tissue and their expression is affected by stage of lactation. J Nutr.

[32] Mihevc SP, Dovč P (2013). Mammary tumors in ruminants. Acta argiculturae Slov.

[33] Choudhary RK, Choudhary S, Pathak D, Verma R. Mucin 1 aberrently expresses n goat mammary carcinoma. 27th Annu. Meet. Indian Soc. Reprod. Fertil. 2017:0072.

[34] Choudhary RK, Choudhary RK, Choudhary S, Verma R (2016). CD10 is a marker of goat mammary Cancer. EC Vet Sci.

[35] Baldassarre H, Deslauriers J, Neveu N, Bordignon V (2011). Detection of endoplasmic reticulum stress markers and production enhancement treatments in transgenic goats expressing recombinant human butyrylcholinesterase. Transgenic Res.

[36] Moh MC, Shen S (2009). The roles of cell adhesion molecules in tumor suppression and cell migration: a new paradox. Cell Adhes Migr.

[37] Chiou S, Wang C, Tseng Y, Lee Y, Chen S, Chou C, et al. A novel role for β 2-microglobulin : a precursor of antibacterial chemokine in respiratory epithelial cells. Sci Rep. 2016:1–12. 10.1038/srep31035 PMC4977529 27503241

[38] Orsi N (2004). The antimicrobial activity of lactoferrin: current status and perspectives. Biometals.

[39] Ginestier C, Hur MH, Charafe-Jauffret E, Monville F, Dutcher J, Brown M (2007). ALDH1 is a marker of normal and malignant human mammary stem cells and a predictor of poor clinical outcome. Cell Stem Cell.

[40] Taylor SC, Laperriere G, Germain H. Droplet Digital PCR versus qPCR for gene expression analysis with low abundant targets: from variable nonsense to publication quality data. Sci Rep. Nature Publishing Group; 2017;7:2409. 10.1038/s41598-017-02217-x PMC5445070 28546538

[41] Baratta M, Chal F (2013). Adults mammary stem cell in cow ’ s Milk : new perspectives and future challenge. J Vet Sci Anim Husb.

[42] Cregan MD, Fan Y, Appelbee A, Brown ML, Klopcic B, Koppen J (2007). Identification of nestin-positive putative mammary stem cells in human breastmilk. Cell Tissue Res.

