# Supplementary material for: Examination of the xanthosine response on gene expression of mammary epithelial cells using RNA-seq technology
Source: J Anim Sci Technol. 2018 Jul 13;60:18. doi: 10.1186/s40781-018-0177-5 (PMC6045846; doi:10.1186/s40781-018-0177-5)
Supplement: Supplementary file 1 — Table S1. List of RT-qPCR primers sequences, product length and their annealing temperatures. Table S2. GO terms (Biological Process, Cellular Components and Molecular Functions) of all expressed genes of goat mammary epithelial cells during early lactation. Table S3. Top 20 transcripts identified by RNA-seq in goat mammary epithelial cells harvested from milk fat layers during early lactation in goat. (DOCX 29 kb) [file 40781_2018_177_MOESM1_ESM.docx]

**Effects of xanthosine on gene expression of mammary epithelial cells using RNA sequencing of goat milk fat globules**

*Shanti Choudhary, Wenli Li^†^, Derek Bickhart, Ramneek Verma, R. S. Sethi, C. S. Mukhopadhay and Ratan K. Choudhary*

*Authors’ address*

*^*^School of Animal Biotechnology, Guru Angad Dev Veterinary and Animal Sciences University, Ludhiana – 101004, Punjab, INDIA*

*^†^Cell Wall Biology and Utilization Research, USDA-ARS, Madison, WI 53706, USA.*

^1^ Corresponding author’s Email: [vetdrrkc@gmail.com](mailto:vetdrrkc@gmail.com)

**Table S1:** List of RT-qPCR primers sequences, product length and their annealing temperatures

| Genes | Forward (F) and reverse (R) primers (5’-3’) | Amplicon length (bp) | Annealing Temperature (ºC) |
| --- | --- | --- | --- |
| *Stem cell/differentiation markers* | | | |
| 1. *HNF4A* | F: GGCAGGAAGGGTATTGGGATGGGA  R: GTGGCTGAGGGAGGCTGGGT | 150 | 60 |
| 1. *ALDH1A1* | F: CTGGGCTGACAAGATCCAGG  R: GACAACCACTGTGTTTCCGC | 172 | 58 |
| 1. *NR5A2* | F: TGACAAAACACAGAGAAAGC  R: AACTTATTCCTTCCTCCGC | 114 | 60 |
| 1. *MSI1* | F: ATGATGCCATGCTGATGTTTG  R: GTTGATTTCGTGGAAGTGGATTTC | 119 | 58 |
| 1. *FNDC3B* | F: ACCACCTACAAGACCCCTCA  R: ACTTCCCACTGACTCGCTTC | 150 | 60 |
| 1. *VIM* | F: GAGAGAGGAAGCCGAGAGCAC  R: CCAGGTCAAGACGTGCCAAA | 131 | 60 |
| 1. *MUC1* | F: ATTGCCCTGGTTGTGTGTCA  R: ATGGGTGTGGTAGGTGGAGT | 111 | 60 |
| 1. *PRB* | F: CAGTGGTCAAGTGGTCTAAATC  R: TCTCCATCCTAGTCCAAATACC | 116 | 57 |
| 1. *ESR1* | F: TTGCTGGCTACTTCGTCTC  R: GGTGGATGTGGTCC(TTCTC | 148 | 57 |
| *Cell proliferation markers* | | | |
| 1. *PCNA* | F: ACGTTGGCACTAGTATTTG  R: CACAGCTATACTCTTGTTCTG | 114 | 60 |
| 1. *TP53* | F: CCCTGTCATCCTTTGTCCCC  R: GGGAATACGTGCAGGTCACA | 108 | 57 |
| *Milk proteins* | | | |
| 1. *CSN2* | F: CAGCAGCAAACAGAGGATGAAC  R: TTTGTGGGAGGCTGTTAGGG | 106 | 60 |
| 1. *LALBA* | F: CCTGAATGGGTCTGTACCGC  R: AGTGAGGGTTCTGGTCGTCT | 136 | 57 |
| *Immune related genes* | | | |
| 1. *TLR4* | F: GGGTGCGGAATGAACTGGTA  R: CTGGGACACCACGACAATCA | 158 | 60 |
| 1. *CXCL14* | F: AACCCTTTCTTTCTCACAGGCA  R: GGCTTGTGATGAAATCTGGAGC | 150 | 58 |
| 1. *CXCR1* | F: TCAGTGTTAAGGCACTCTGGTCT  R: TCCTCAAACCCCTCCCACAAAT | 149 | 60 |
| *Differentially expressed genes* | | | |
| 1. *FOS* | F: TGGACTCAAGTCCTTACCTCT  R: GGCTCCACATGCTACTAACTAC | 103 | 60 |
| 1. *JUNB* | F: AAATGGAACAGCCCTTCTACC  R:  TGGGTTTCAGGAGTTTGTAGTC | 102 | 60 |
| 1. *PECAM1* | F: GAGTATGAGGTGTGGGTGAAAG  R: CTGGGACAGAACAGTTGACTAC | 103 | 60 |
| 1. *LTF* | F:  GGAAGGCACAGGAGAAGTTT  R:  CCAAGGGCAGAGTCTTTGAATA | 103 | 60 |
| 1. *SELL* | F: TGCTGTGCCTGATGACATATAC  R:  ACTGGAAGGACTAGCGAGATAA | 109 | 60 |
| 1. *THBS1* | F: TCAGGGCAGGAAGACTATGA  R: ATACTGGGCTGGGTTGTAATG | 120 | 60 |
| *Endogenous control* | | | |
| 1. *RPL4* | F: TTGGAAACATGTGTCGTGGG  R: GCAGATGGCGTATCGCTTCT | 101 | 60 |
| 1. *RPS23* | F: CCCAATGATGGTTGCTTGAA  R: CGGACTCCAGGAATGTCACC | 101 | 58 |

**Table S2**: GO terms (Biological Process, Cellular Components and Molecular Functions) of all expressed genes of goat mammary epithelial cells during early lactation.

| GO Term: Biological Process | # of genes |
| --- | --- |
| cellular process (GO:0009987) | 3587 |
| metabolic process (GO:0008152) | 3495 |
| localization (GO:0051179) | 1023 |
| cellular component organization or biogenesis (GO:0071840) | 886 |
| response to stimulus (GO:0050896) | 739 |
| biological regulation (GO:0065007) | 649 |
| developmental process (GO:0032502) | 600 |
| multicellular organismal process (GO:0032501) | 391 |
| immune system process (GO:0002376) | 294 |
| biological adhesion (GO:0022610) | 122 |
| reproduction (GO:0000003) | 97 |
| locomotion (GO:0040011) | 46 |
| growth (GO:0040007) | 3 |
| rhythmic process (GO:0048511) | 2 |
| cell killing (GO:0001906) | 2 |
|  |  |
| *GO Terms: Cellular Components* |  |
| cell part (GO:0044464) | 2334 |
| organelle (GO:0043226) | 1489 |
| macromolecular complex (GO:0032991) | 902 |
| membrane (GO:0016020) | 637 |
| extracellular region (GO:0005576) | 113 |
| cell junction (GO:0030054) | 45 |
| extracellular matrix (GO:0031012) | 40 |
|  |  |
| *GO Terms: Molecular Functions* |  |
| catalytic activity (GO:0003824) | 2655 |
| binding (GO:0005488) | 2375 |
| structural molecule activity (GO:0005198) | 424 |
| transporter activity (GO:0005215) | 359 |
| receptor activity (GO:0004872) | 204 |
| signal transducer activity (GO:0004871) | 80 |
| translation regulator activity (GO:0045182) | 58 |
| antioxidant activity (GO:0016209) | 16 |
| channel regulator activity (GO:0016247) | 5 |
| catalytic activity (GO:0003824) | 2655 |
| binding (GO:0005488) | 2375 |
| structural molecule activity (GO:0005198) | 424 |

**Table S3:** Top 20 transcripts identified by RNA-seq in goat mammary epithelial cells harvested from milk fat layers during early lactation in goat.

| CON_741R | |  | TRT_741L | |  | CON_647R | |  | TRT_647L | |
| --- | --- | --- | --- | --- | --- | --- | --- | --- | --- | --- |
| Gene | FPKM |  | Gene | FPKM |  | Gene | FPKM |  | Gene | FPKM |
| CSN3 | 39234.7 |  | CSN3 | 40343.9 |  | LALBA | 50756 |  | LOC108634775 | 190458 |
| LALBA | 37817.3 |  | LALBA | 38730.6 |  | PAEP | 39768.8 |  | PAEP | 59875 |
| PAEP | 36319.2 |  | PAEP | 36616.8 |  | CSN3 | 39338.3 |  | CSN2 | 48175.7 |
| PLIN2 | 11204.3 |  | PLIN2 | 14890.3 |  | LOC108634775 | 9376.33 |  | CSN1S1 | 41731 |
| LOC108634775 | 9166.94 |  | GLYCAM1 | 7894.63 |  | GLYCAM1 | 8048.68 |  | CSN3 | 27781.3 |
| GLYCAM1 | 8169.76 |  | TPT1 | 7235.37 |  | TPT1 | 7986.15 |  | LALBA | 24821.9 |
| TPT1 | 6314.23 |  | RPS29 | 4725.97 |  | RPS29 | 5632.76 |  | CSN1S2 | 21949.6 |
| RPLP1 | 3969.04 |  | RPLP1 | 4276.38 |  | RPLP1 | 5151.65 |  | PLIN2 | 6679.86 |
| RPS29 | 3917.86 |  | RPS8 | 3758.55 |  | RPS8 | 4711.61 |  | RPLP1 | 5597.26 |
| RPS8 | 3378.21 |  | RPL23 | 3680.28 |  | RPL23 | 4631.97 |  | RPS29 | 5289.1 |
| RPS2 | 3294.6 |  | RPL13A | 3591.93 |  | RPL21 | 4476.09 |  | RPS2 | 5056.37 |
| RPL13A | 3272.78 |  | RPS17 | 3402.26 |  | PLIN2 | 4301.92 |  | TPT1 | 4644.13 |
| RPL23 | 3199.28 |  | RPL21 | 3290.45 |  | RPL13A | 4127.22 |  | RPS8 | 4232.62 |
| FABP3 | 3097.79 |  | RPS24 | 3274.98 |  | RPS2 | 4092.48 |  | FABP3 | 4068.23 |
| RPS17 | 2971.79 |  | RPS2 | 3188.25 |  | RPL31 | 4065.09 |  | RPS9 | 3996.27 |
| RPL21 | 2943.12 |  | RPL27A | 3127.26 |  | FABP3 | 3873.75 |  | GLYCAM1 | 3953.21 |
| RPL31 | 2730.54 |  | RPL31 | 3071.65 |  | RPS17 | 3749.45 |  | RPS10 | 3811.01 |
| RPL27A | 2703.09 |  | FABP3 | 2955.48 |  | RPL27A | 3528.59 |  | RPL19 | 3717.74 |
| RPS12 | 2695.7 |  | RPS12 | 2842.12 |  | RPS24 | 3296.78 |  | RPL8 | 3705.42 |
| RPS24 | 2649.12 |  | RPS18 | 2826.92 |  | RPS18 | 3165.33 |  | RPS17 | 3596.8 |
